# Supplementary material for: The Effectiveness of Nutrition Interventions Combined with Exercise in Upper Gastrointestinal Cancers: A Systematic Review
Source: Nutrients. 2021 Aug 18;13(8):2842. doi: 10.3390/nu13082842 (PMC8400981; doi:10.3390/nu13082842)
Supplement: Supplementary file 1 [file nutrients-13-02842-s001.zip › nutrients-1329505-supplementary.pdf]

The effectiveness of nutrition interventions combined with exercise in upper gastrointestinal cancer- a systematic review

SUPPLEMENTARY MATERIAL

Contents

S1.Search Terms 2

S2.Details of Relevant Ongoing Clinical Trials.. 7

S1. Search Terms

Data Base	Search Strategy
EMBASE	1.'digestive system tumor cell line'/exp OR 'digestive system tumor'/exp<br>2.((oesophag* OR esophag* OR gastroesophag*) NEAR/3 (cancer* OR neoplasm* OR tumo?r* OR adenocarcinoma* OR carcinoma*)):ab,ti<br>3.((stomach OR gastric) NEAR/3 (cancer* OR neoplasm* OR tumo?r* OR adenocarcinoma* OR carcinoma*)):ab,ti<br>4.(('bile duct*' OR gallbladder* OR biliary OR liver OR hepato* OR hepatic) NEAR/3 (cancer* OR neoplasm* OR tumo?r* OR adenocarcinoma* OR carcinoma*)):ab,ti<br>5.(pancrea* NEAR/3 (cancer* OR neoplasm* OR tumo?r* OR adenocarcinoma* OR carcinoma*)):ab,ti<br>6.((‘upper gi’ OR ‘upper gastroinstestinal’) NEAR/3 (cancer* OR neoplasm* OR tumo?r* OR adenocarcinoma* OR carcinoma*)):ab,ti<br>7.#1 OR #2 OR #3 OR #4 OR #5 OR #6<br>8.'nutrition education'/exp OR 'diet therapy'/exp OR 'cooking'/exp OR 'nutrition'/exp OR 'enteric feeding'/exp OR 'parenteral nutrition'/exp OR 'nutritional support'/exp OR 'nutrition service'/exp OR 'cachexia'/exp<br>9.(Nutrition* NEAR/3 (intervention* OR therap* OR education OR support* OR treatment* OR consultation? OR consult? OR cancer* OR counsel*)):ti,ab<br>10.((diet? OR dietary) NEAR/3 (intervention* OR therap* OR education OR support* OR treatment* OR consultation? OR consult? OR cancer* OR counsel* OR restrict*)):ti,ab<br>11.(Parenteral NEAR/3 (alimentation OR feed* OR fluid OR hyperalimentation)):ti,ab<br>12.(Nutrition* NEAR/3 (product* OR supplement* OR support* OR mixture)):ti,ab<br>13.(Diet* NEAR/3 (product* OR supplement* OR support* OR mixture)):ti,ab<br>14.((Diet* OR nutrition*) NEAR/5 (educat* OR guideline*)):ti,ab<br>15.#8 OR #9 OR #10 OR #11 OR #12 OR #13 OR #14<br>16.'exercise'/exp OR 'kinesiotherapy'/exp OR 'physical activity'/exp OR 'physical activity, capacity and performance'/de OR 'training'/de OR 'endurance'/de OR 'exercise tolerance'/de OR 'physical capacity'/de OR 'sport'/exp<br>17.(exercis* OR isometric* or isotonic* or isokinetic*):ti,ab<br>18.((resistance OR strength*) NEAR/3 train*):ti,ab<br>19.((physical* or motion* or cardiopulmonary or cardiorespiratory) NEAR/3 (fit* or therap*)):ti,ab<br>20.(treadmill* or cross-train* or rowing or sport* OR exercise* OR "physical activit*" OR aerobic* OR run or jog* or running OR walk or walks or walking OR gym* OR yoga oR pilates OR "recreation* activit*" OR zumba or salsa* OR cycling or bicycle or bike or swim* or dance or dancer* or dances or dancing or HIIT):ti,ab<br>21.(circuit* NEAR/1 train*):ti,ab<br>22.(keep* NEAR/1 (active or fit)):ti,ab

	23.#16 OR #17 OR #18 OR #19 OR #20 OR #21 OR #22<br>24.#7 AND #15 AND #23<br>25.'conference abstract':it OR 'conference review':it OR 'editorial':it OR 'letter':it<br>26.#24 NOT #25
Medline (OVID)	1.exp Esophageal Neoplasms/ OR Stomach Neoplasms/ OR exp Biliary Tract Neoplasms/ or exp Liver Neoplasms/ or exp Pancreatic Neoplasms/ OR Gastrointestinal Neoplasms/<br>2.((oesophag* OR esophag* OR gastroesophag*) ADJ3 (cancer* OR neoplasm* OR tumo?r* OR adenocarcinoma* OR carcinoma*)).ti,ab.<br>3.((stomach OR gastric) ADJ3 (cancer* OR neoplasm* OR tumo?r* OR adenocarcinoma* OR carcinoma*)).ti,ab.<br>4.((bile duct* OR gallbladder* OR biliary OR liver OR hepato* OR hepatic) ADJ3 (cancer* OR neoplasm* OR tumo?r* OR adenocarcinoma* OR carcinoma*)).ti,ab.<br>5.(pancrea* ADJ3 (cancer* OR neoplasm* OR tumo?r* OR adenocarcinoma* OR carcinoma*)).ti,ab.<br>6.((upper gi OR upper gastroinstestinal) ADJ3 (cancer* OR neoplasm* OR tumo?r* OR adenocarcinoma* OR carcinoma*)).ti,ab.<br>7.or/1-6<br>8.exp Nutrition Therapy/ OR Nutrition Assessment/ OR Cachexia/ OR Diet Records/ OR exp Nutrition Surveys/ OR Nutritional Status/ OR Dietetics/ OR Nutrition Policy/<br>9.(Nutrition* ADJ3 (intervention* OR therap* OR education OR support* OR treatment* OR consultation? OR consult? OR cancer* OR counsel*)).ti,ab.<br>10.((diet? OR dietary) ADJ3 (intervention* OR therap* OR education OR support* OR treatment* OR consultation? OR consult? OR cancer* OR counsel* OR restrict*)).ti,ab.<br>11.(Parenteral ADJ3 (alimentation OR feed* OR fluid OR hyperalimentation)).ti,ab.<br>12.(Nutrition* ADJ3 (product* OR supplement* OR support* OR mixture)).ti,ab.<br>13.(Diet* ADJ3 (product* OR supplement* OR support* OR mixture)).ti,ab.<br>14.((Diet* OR nutrition*) ADJ5 (educat* OR guideline* OR factor*)).ti,ab.<br>15.or/8-14<br>16.exp Exercise/ OR exp Exercise Therapy/ OR exp Physical Fitness/ OR exp "physical education and training"/ OR exp "Exercise Movement Techniques"/ or physical endurance/ or exercise tolerance/ OR Physical Exertion/ or exp Sports/ or Dancing/<br>17.(isometric* or isotonic* or isokinetic* or exercis*).ti,ab.<br>18.((resistance OR strength) adj3 (train* OR exercise*)).ti,ab.<br>19.((physical* or motion* or cardiopulmonary or cardiorespiratory) adj3 (fit* or therap* or activit*)).ti,ab.<br>20.(treadmill* or cross-train* or rowing or sport* or exercise* or physical* activit* or aerobic* or run or jog* or running or walk or walks or walking or gym* or yoga or pilates or "recreation* activit*" or zumba or salsa* or cycling or bicycle or bike or swim* or dance or dancer* or dances or dancing or physiotherapy* or physical therap*).ti,ab.<br>21.(circuit* adj1 train*).ti,ab.

	22.(keep* adj1 (active or fit)).ti,ab.<br>23.or/16-22<br>24.7 AND 15 AND 23
Web of Science	TS =((((oesophag* OR esophag* OR gastroesophag*) NEAR/2 (cancer* OR neoplasm* OR tumo*r* OR adenocarcinoma* OR carcinoma*)) OR ((stomach OR gastric) NEAR/2 (cancer* OR neoplasm* OR tumo*r* OR adenocarcinoma* OR carcinoma*)) OR ((“bile duct*” OR gallbladder* OR biliary OR liver OR hepato* OR hepatic) NEAR/2 (cancer* OR neoplasm* OR tumo*r* OR adenocarcinoma* OR carcinoma*)) OR (pancrea* NEAR/2 (cancer* OR neoplasm* OR tumo*r* OR adenocarcinoma* OR carcinoma*)) OR ((“upper gi” OR “upper gastroinstestinal”) NEAR/2 (cancer* OR neoplasm* OR tumo*r* OR adenocarcinoma* OR carcinoma*))) AND ((Nutrition* NEAR/2 (intervention* OR therap* OR education OR support* OR treatment* OR consultation* OR consult* OR cancer* OR counsel*)) OR ((diet* OR dietary) NEAR/2 (intervention* OR therap* OR education OR support* OR treatment* OR consultation* OR consult* OR cancer* OR counsel* OR restrict*)) OR (Parenteral NEAR/2 (alimentation OR feed* OR fluid OR hyperalimentation)) OR (Nutrition* NEAR/2 (product* OR supplement* OR support* OR mixture)) OR (Diet* NEAR/2 (product* OR supplement* OR support* OR mixture)) OR ((Diet* OR nutrition*) NEAR/5 educat*)) AND ((exercis* OR isometric* or isotonic* or isokinetic*) OR ((resistance OR strength*) NEAR/2 train*) OR ((physical* or motion* or cardiopulmonary or cardiorespiratory) NEAR/2 (fit* or therap*)) OR (treadmill* or cross-train* or rowing or sport* OR exercise* OR "physical activit*" OR aerobic* OR run or jog* or running OR walk or walks or walking OR gym* OR yoga OR pilates OR "recreation* activit*" OR zumba or salsa* OR cycling or bicycle or bike or swim* or dance or dancer* or dances or dancing or HIIT) OR (circuit* NEAR/1 train*) OR (keep* NEAR/1 (active or fit))))
CINAHL	1.(MH "Biliary Tract Neoplasms+") OR (MH "Liver Neoplasms+") OR (MH "Pancreatic Neoplasms+") OR (MH "Esophageal Neoplasms+") OR (MH "Stomach Neoplasms")<br>2.TI ((oesophag* OR esophag* OR gastroesophag*) N2 (cancer* OR neoplasm* OR tumo#r* OR adenocarcinoma* OR carcinoma*)) OR AB ((oesophag* OR esophag* OR gastroesophag*) N2 (cancer* OR neoplasm* OR tumo#r* OR adenocarcinoma* OR carcinoma*))<br>3.TI ((stomach OR gastric) N2 (cancer* OR neoplasm* OR tumo#r* OR adenocarcinoma* OR carcinoma*)) OR AB ((stomach OR gastric) N2 (cancer* OR neoplasm* OR tumo#r* OR adenocarcinoma* OR carcinoma*))<br>4.TI ((“bile duct*” OR gallbladder* OR biliary OR liver OR hepato* OR hepatic) N2 (cancer* OR neoplasm* OR tumo#r* OR adenocarcinoma* OR carcinoma*)) OR AB ((“bile duct*” OR gallbladder* OR biliary OR liver OR hepato* OR hepatic) N2 (cancer* OR neoplasm* OR tumo#r* OR adenocarcinoma* OR carcinoma*))<br>5.TI (pancrea* N2 (cancer* OR neoplasm* OR tumo#r* OR adenocarcinoma* OR carcinoma*)) OR AB (pancrea* N2 (cancer* OR neoplasm* OR tumo#r* OR adenocarcinoma* OR carcinoma*))<br>6.TI ((“upper gi” OR “upper gastroinstestinal”) N2 (cancer* OR neoplasm* OR tumo#r* OR adenocarcinoma* OR carcinoma*)) OR AB ((“upper gi” OR “upper gastroinstestinal”) N2 (cancer* OR neoplasm* OR tumo#r* OR adenocarcinoma* OR carcinoma*))<br>7.S1 OR S2 OR S3 OR S4 OR S5 OR S6<br>8.MH "Nutrition Education" OR MH "Nutritional Status" OR MH "Food Habits" OR MH "Diet" OR MH "Nutritional Counseling" OR MH "Nutritional Assessment" OR MH "Nutritional Requirements" OR MH "Nutritional Support" OR MH "Parenteral Nutrition" OR MH "Enteral Nutrition" OR MH "Dietary Supplements" OR MH "Cachexia"<br>9.TI (Nutrition* N2 (intervention* OR therap* OR education OR support* OR treatment* OR consultation# OR consult# OR cancer* OR counsel*)) OR AB (Nutrition* N2 (intervention* OR therap* OR education OR support* OR treatment* OR consultation# OR consult# OR cancer* OR counsel*))

	10.TI ((diet# OR dietary) N2 (intervention* OR therap* OR education OR support* OR treatment* OR consultation# OR consult* OR cancer* OR counsel* OR restrict*)) OR AB ((diet# OR dietary) N2 (intervention* OR therap* OR education OR support* OR treatment* OR consultation# OR consult* OR cancer* OR counsel* OR restrict*))<br>11.TI (Parenteral N2 (alimentation OR feed* OR fluid OR hyperalimentation)) OR AB (Parenteral N2 (alimentation OR feed* OR fluid OR hyperalimentation))<br>12.TI (Nutrition* N2 (product* OR supplement* OR support* OR mixture)) OR AB (Nutrition* N2 (product* OR supplement* OR support* OR mixture))<br>13.TI (Diet* N2 (product* OR supplement* OR support* OR mixture)) OR AB (Diet* N2 (product* OR supplement* OR support* OR mixture))<br>14.TI ((Diet* OR nutrition*) N4 (educat*OR guideline* OR factor*)) OR AB ((Diet* OR nutrition*) N4 (educat* OR guideline* OR factor*))<br>15.S9 OR S10 OR S11 OR S12 OR S13 OR S14 OR S15 OR S16<br>16.(MH "Exercise+") OR (MH "Physical Activity") OR (MH "Physical Fitness+") OR (MH "Physical Performance") OR (MH "Sports+") OR (MH "Resistance Training") OR (MH "Therapeutic Exercise+") OR (MH "Exercise Intensity")<br>17.TI (isometric* or isotonic* or isokinetic* OR exercis*) OR AB isometric* or isotonic* or isokinetic* OR exercis*)<br>18.TI ((resistance OR strength) N3 (train* OR exercis*)) OR AB ((resistance OR strength) N3 (train* OR exercis*))<br>19.TI ((physical* or motion* or cardiopulmonary or cardiorespiratory) N3 (fit* or therap*)) OR AB ((physical* or motion* or cardiopulmonary or cardiorespiratory) N3 (fit* or therap*))<br>20.TI (treadmill* or cross-train* or rowing or sport* OR exercise* OR "physical activit*" OR aerobic* OR run or jog* or running OR walk or walks or walking OR gym* OR yoga oR pilates OR "recreation* activit*" OR zumba or salsa* OR cycling or bicycle or bike or swim* or dance or dancer* or dances or dancing) OR AB (treadmill* or cross-train* or rowing or sport* OR exercise* OR "physical activit*" OR aerobic* OR run or jog* or running OR walk or walks or walking OR gym* OR yoga oR pilates OR "recreation* activit*" OR zumba or salsa* OR cycling or bicycle or bike or swim* or dance or dancer* or dances or dancing)<br>21.TI (circuit* N1 train*) OR AB (circuit* N1 train*)<br>22.TI (keep* N1 (active or fit)) OR AB (keep* N1 (active or fit))<br>23.S16 OR S17 OR S18 OR S19 OR S20 OR S21 OR S22<br>24.S7 AND S15 AND S23
Cochrane Library	1.[mh “Esophageal Neoplasms”] OR [mh “Stomach Neoplasms”] OR [mh ” Biliary Tract Neoplasms”] OR [mh “Liver Neoplasms”] OR [mh “Pancreatic Neoplasms”]<br>2.((oesophag* OR esophag* OR gastroesophag*) NEAR/3 (cancer* OR neoplasm* OR tumo*r* OR adenocarcinoma* OR carcinoma*)):ti,ab,kw<br>3.((stomach OR gastric) NEAR/3 (cancer* OR neoplasm* OR tumo*r* OR adenocarcinoma* OR carcinoma*)):ti,ab,kw<br>4.((“bile duct*” OR gallbladder* OR biliary OR liver OR hepato* OR hepatic) NEAR/3 (cancer* OR neoplasm* OR tumo*r* OR adenocarcinoma* OR carcinoma*)):ti,ab,kw<br>5.(pancrea* NEAR/3 (cancer* OR neoplasm* OR tumo*r* OR adenocarcinoma* OR carcinoma*)):ti,ab,kw<br>6.((“upper gi” OR “upper gastroinstestinal”) NEAR/3 (cancer* OR neoplasm* OR tumo*r* OR adenocarcinoma* OR carcinoma*)):ti,ab,kw<br>7.#1 OR #2 OR #3 OR #4 OR #5 OR #6<br>8.[mh “Nutrition Therapy”] OR [mh “Nutrition Assessment”] OR [mh “Cachexia”] OR [mh “Diet Records”] OR [mh “Nutrition Surveys”] OR [mh “Nutritional Status”] OR [mh “Dietetics”] OR [mh “Nutrition Policy”]<br>9.(Nutrition* NEAR/3 (intervention* OR therap* OR education OR support* OR treatment* OR consultation* OR consult* OR cancer* OR counsel*)):ti,ab,kw

	10.((diet* OR dietary) NEAR/3 (intervention* OR therap* OR education OR support* OR treatment* OR consultation* OR consult* OR cancer* OR counsel* OR restrict*)):ti,ab,kw<br>11.(Parenteral NEAR/3 (alimentation OR feed* OR fluid OR hyperalimentation)):ti,ab,kw<br>12.(Nutrition* NEAR/3 (product* OR supplement* OR support* OR mixture)):ti,ab,kw<br>13.(Diet* NEAR/3 (product* OR supplement* OR support* OR mixture)):ti,ab,kw<br>14.((Diet* OR nutrition*) NEAR/5 (educat*OR guideline*)):ti,ab,kw<br>15.#8 OR #9 OR #10 OR #11 OR #12 OR #13 OR #14<br>16.[mh “Exercise”] OR [mh “Exercise Therapy”] OR [mh “Physical Fitness”] OR [mh "physical education and training"] OR [mh "Exercise Movement Techniques"] or [mh “physical endurance”] or [mh “exercise tolerance”] OR [mh “Physical Exertion”] or [mh “Sports”] or [mh “Dancing”]<br>17.(isometric* or isotonic* or isokinetic* OR exercis*):ti,ab,kw<br>18.((resistance OR strength) NEAR/3 (train* OR exercise*)):ti,ab,kw<br>19.((physical* or motion* or cardiopulmonary or cardiorespiratory) NEAR/3 (fit* or therap*)):ti,ab,kw<br>20.(treadmill* or cross-train* or rowing or sport* OR exercise* OR "physical activit*" OR aerobic* OR run or jog* or running OR walk or walks or walking OR gym* OR yoga oR pilates OR "recreation* activit*" OR zumba or salsa* OR cycling or bicycle or bike or swim* or dance or dancer* or dances or dancing):ti,ab,kw<br>21.(circuit* NEAR/1 train*):ti,ab,kw<br>22.(keep* NEAR/1 (active or fit)):ti,ab,kw<br>23.#16 OR #17 OR #18 OR #19 OR #20 OR #21 OR #22<br>24.#7 AND #15 AND #23

S2. Details of Relevant Ongoing Clinical Trials

Author (PI)	Title	Clinical Trial Registry	Registration ID	URL
Shinnno Naoki	Randomized trial of Exercise and Nutritional prehabilitation for Elderly Gastric Cancer patients	Japan Registry of Clinical Trials	jRCTs051190015	https://rctportal.niph.go.jp/en/detail?trial_id=jRCTs051190015
Hiroshi MIyata	Rehabilitation / nutrition intervention trial for elderly patients with oesophageal cancer	Japan Registry of Clinical Trials	jRCTs051190016	https://rctportal.niph.go.jp/en/detail?trial_id=jRCTs051190016
Sophie Allen	A randomised controlled trial to assess whether prehabilitation improves fitness in patients undergoing neoadjuvant treatment prior to oesophagogastric cancer surgery : study protocol	ClinicalTrials.gov	NCT02950324	https://pubmed.ncbi.nlm.nih.gov/30580268/
Li Yiran	A comprehensive intervention to improve quality of life in operated esophageal cancer patients: a randomized controlled trial	Chinese Clinical Trial Registry	ChiCTR1800019823	http://www.chictr.org.cn/showprojen.aspx?proj=32884
Bertrand Le Roy	effect of prehabilitation in gastrooesophageal adenocarcinoma: study protocol of a multicentric, randomised, control trial—the PREHAB study	ClinicalTrials.gov	NCT02780921	https://pubmed.ncbi.nlm.nih.gov/27927660/
Yu-Juan Xu	Effects of a "Walk, Eat, & Breathe" Nursing Intervention for Patients With Esophageal Cancer	ClinicalTrials.gov	NCT02850172	https://clinicaltrials.gov/ct2/show/NCT02850172
Augustinas Bausys	Prehabilitation for Gastrectomy (PREFOG)	ClinicalTrials.gov	NCT04223401	https://clinicaltrials.gov/ct2/show/NCT04223401
Author (PI)	Title	Clinical Trial Registry	Registration ID	URL

M. Sosef	Preoperative training for patients with esophageal cancer who will undergo resection (PC-OCR II)	Netherlands Trial Register	NTR3351	https://www.trialregister.nl/trial/3200
Stefano Turi	Prehabilitation in Esophageal Surgery (PRESS)	ClinicalTrials.gov	NCT03798951	https://clinicaltrials.gov/ct2/show/NCT03798951
Makoto Yamasaki	Randomized controlled study on the efficacy of the exercise and amino acid for the elderly with esop hageal squamous cell carcinoma during neoadjuvant chemotherapy	The University Hospital Medical Information Network Center	UMIN000034505	https://upload.umin.ac.jp/cgi-open-bin/ctr_e/ctr_view.cgi?recptno=R000039340
Susumu Aikou	Randomized controlled trials on prevention of sarcopenia during gastric cancer surgery by exercise and nutrition intervention	The University Hospital Medical Information Network Center	UMIN000033613	https://upload.umin.ac.jp/cgi-open-bin/ctr_e/ctr_view.cgi?recptno=R000038333
Linda O’Neill	Rehabilitation strategies following oesophagogastric and Hepatopancreaticobiliary cancer (ReStOre II): a protocol for a randomized controlled tria	ClinicalTrials.gov	NCT03958019	https://bmccancer.biomedcentral.com/articles/10.1186/s12885-020-06889-z
